# Feasibility of Detecting Pulmonary Embolism Using Noncontrast MRI

**DOI:** 10.5402/2013/729271

**Published:** 2012-11-28

**Authors:** C. S. Mudge, T. T. Healey, M. K. Atalay, J. A. Pezzullo

**Affiliations:** Department of Diagnostic Imaging, Rhode Island Hospital, 593 Eddy Street, Providence, RI 02903, USA

## Abstract

*Purpose*. The purpose of this study was to evaluate the feasibility of detecting pulmonary emboli utilizing noncontrast magnetic resonance imaging techniques in patients with known pulmonary embolism. *Materials and Methods*. Eleven patients were enrolled in a study to evaluate right ventricular function by cardiac MRI in patients diagnosed with acute pulmonary embolism on CT pulmonary angiogram. Cardiac MRI was performed as soon as possible following pulmonary embolism detection. Two independent observers reviewed the precontrast portion of each MRI, scoring right, left, and lobar arteries as positive or negative for PE. The CTs were reviewed and interpreted in the same manner. *Results*. MRI was obtained on average of 40 hours after the CT. Forty-eight vessels were affected by PE on CT, 69% of which were identified on MRI. All eight pulmonary emboli located in the right or left pulmonary arteries were detected on MRI. Of the 15 pulmonary emboli that were not detected on MRI, 7 were subsegmental, 6 were segmental, and 2 were located in a branch not included in the MRI field of view. *Conclusions*. Most pulmonary emboli detected on CT were identified on noncontrast MRI, even though our MRI protocol was not optimized for pulmonary artery visualization.

## 1. Introduction 

Pulmonary embolism (PE) is a serious condition responsible for significant morbidity and mortality. PE is currently the third leading cause of cardiovascular death in the United States [1]. PE requires prompt diagnosis and treatment to prevent potentially deadly consequences. Currently, CT pulmonary angiography (CTPA) is considered the gold standard in the making of the diagnosis [2, 3]. However, CTPA requires the use of iodinated contrast media with its risk of renal failure and ionizing radiation with its risk of cancer induction [4, 5]. Many patients with suspected PE, such as pregnant women and patients with impaired renal function, have at least a relative contraindication to contrast media or radiation. 

MRI offers a potential alternative to CTPA in the evaluation of the pulmonary vasculature and the diagnosis of PE [6–8]. To date, however, the majority of studies evaluating the use of MRI in the diagnosis of PE have used gadolinium-based intravenous contrast media, which is contraindicated in pregnant patients and in those with renal failure [9]. The purpose of this study was to evaluate the feasibility of detecting pulmonary emboli using noncontrast bright blood and dark blood MR imaging techniques. 

## 2. Materials and Methods 

### 2.1. Subjects

This HIPAA-compliant study was approved by our institutional review board. Eligible patients included those diagnosed with PE on CTPA. Exclusion criteria included any contraindication to MRI or gadolinium-based contrast media. All patients were already enrolled in a prospective study to evaluate right ventricular function by cardiac MR (CMR) in patients diagnosed with acute PE on CT pulmonary angiogram (CTPA), with informed written consent obtained at the time of study enrollment. The final study population comprised 11 patients (5 males, 6 females) with an average age (±SD) of 56.6 ± 10.8 years (age range, 45–78 years). 

### 2.2. CT

Four different CT scanners were used with the protocols outlined below. 

Four studies were conducted on a 16-row CT scanner (Sensation 16, Siemens Medical Solutions, Erlangen, Germany). With the use of helical acquisition, patients were scanned supine in a caudocranial direction with a scan range based on the scout view from 1 cm below the lowest costophrenic angle to 1 cm above the lung apex. This range was intended to completely cover the lungs and to accommodate any slight changes in patient positioning or breath holding between the scout view and the CTPA. Scan parameters were as follows: in-plane field of view FOV, 38 cm; matrix, 512 × 512; pitch, 1.25; collimation, 1.5 mm; rotation time, 0.75 second; tube voltage, 120 kVp and tube current time product, 220 mA. Images were reconstructed at 2 mm slice thickness with no overlap. For CTPA, 100 mL of iopamidol 370 mg I/mL (Isovue General Electric Healthcare, Waukesha, WI, USA) was injected intravenously at 4 mL/s with the use of a dual-head power injector (Stellant D Medrad, Indianola, PA, USA) after a fixed scan delay of 22 seconds. 

Two studies were conducted on a 64-row CT scanner (Light Speed VCT General Electric Healthcare). With the use of helical acquisition, patients were scanned supine in a caudocranial direction from 1 cm below the lowest costophrenic angle to 1 cm above the lung apex. Scan parameters were as follows: FOV, 38 cm; matrix, 512 × 512; pitch, 1.375; collimation, 0.625 mm; rotation time, 0.8 second; tube voltage, 100 kVp; tube current, 100–750 mA, set to a noise index of 40.0. Images were reconstructed at 2.5 mm slice thickness with no overlap. For CTPA, 100 mL of iopamidol 370 mg I/mL was injected intravenously at 4 mL/s with the use of a dual-head power injector after a fixed scan delay of 22 seconds. 

One study was conducted on a 16-row CT scanner (Light Speed 16, General Electric Healthcare). With the use of helical acquisition, the patient was scanned supine in a craniocaudal direction from 1 cm above the lung apex to 1 cm below the lowest costophrenic angle. Scan parameters were as follows: FOV, 38 cm; matrix, 512 × 512; pitch, 1.75; collimation, 1.25 mm; rotation time, 0.5 seconds; tube voltage, 120 kVp; tube current, 100–440 mA, set to a noise index of 24. Images were reconstructed at 2.5 mm slice thickness with no overlap. For CTPA, 100 mL of iopamidol 370 mg I/mL was injected intravenously at 4 mL/s with the use of a dual-head power injector after a fixed scan delay of 22 seconds. 

Four studies were conducted on a 4-row CT scanner (Light Speed QX/I, General Electric Healthcare). With the use of helical acquisition, the patient was scanned supine in a craniocaudal direction from 1 cm above the lung apex to 1 cm below the lowest costophrenic angle. Scan parameters were as follows: FOV, 38 cm; matrix, 512 × 512; pitch, 1.5; collimation, 2.5 mm; rotation time, 0.8 seconds; tube voltage, 120 kVp; tube current, 80–440 mA, set to a noise index of 15.86. Images were reconstructed at 2.5 mm slice thickness with no overlap. For CTPA, 120 mL of iohexol 350 mg/mL (Omnipaque 350, General Electric Healthcare, Waukesha, WI, USA) was injected intravenously at 4 mL/s with the use of a dualhead power injector after a fixed scan delay of 20 seconds. 

CT image acquisition took on average (±SD) 2.7 ± 0.9 minutes.

Images were reviewed at a PACS workstation picture archiving and communication system workstation (PACS, Centricity Radiology RA1000; General Electric Healthcare), where windows and levels could be adjusted by the reviewer. 

### 2.3. MRI

MRI was obtained an average (±SD) of 39.9 ± 26.2 hours after the CTPA (range 19.3–109.4 hours). MRI image acquisition took on average (±SD) 55.6 ± 6.8 minutes. CMR was performed on a Siemens Symphony 1.5 T system. All studies were performed on a 1.5-T MRI Siemens Symphony scanner (Siemens Medical Systems, Malvern, PA, USA) equipped with Quantum gradients. Subjects were imaged in the supine position using a six-element phased-array chest coil. After scout imaging was completed, transaxial cine imaging was conducted using SSFP (echo time, 1.4–2.3 ms; repetition time, 48–53 ms; flip angle 40–45°; FOV 26–35 cm; matrix, 156 × 192) with retrospective ECG gating. Data were acquired during end-inspiration. In all cases, slice thickness was 8 mm and slice separation was 2 mm. Twenty-five cardiac phases were reconstructed for each cine loop. The *z*-axis field of view extended from the midaortic arch to the inferior margin of the heart. Images were reviewed at a PACS workstation, where windows and levels could be adjusted by the reviewer. Although additional imaging was conducted using different pulse sequences, imaging orientations, and gadolinium-based intravenous contrast, only the precontrast axial cine series were evaluated in this study. 

### 2.4. Data Analysis

Two independent observers reviewed each MRI examination. Each main and lobar pulmonary artery was scored as positive or negative for pulmonary embolism. After this analysis was completed, the reviewers jointly evaluated the CTPA studies, which were used as the gold standard. 

### 2.5. Statistical Analysis

Continuous variables are reported as means (±SD). Interobserver agreement was evaluated using the *κ* statistic.

## 3. Results 

**Figure 1:**
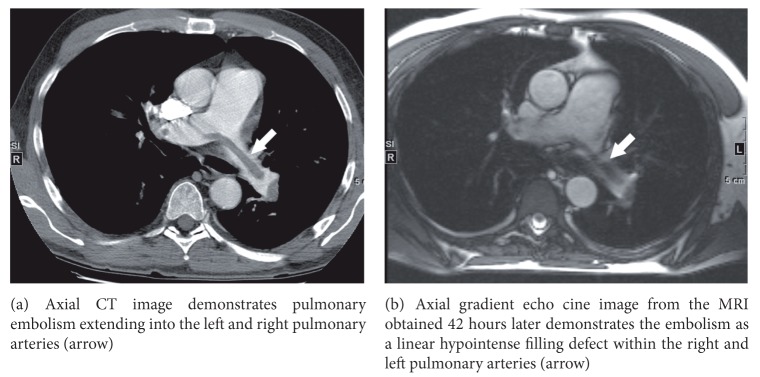
59-year-old male with a saddle pulmonary embolus.

**Figure 2:**
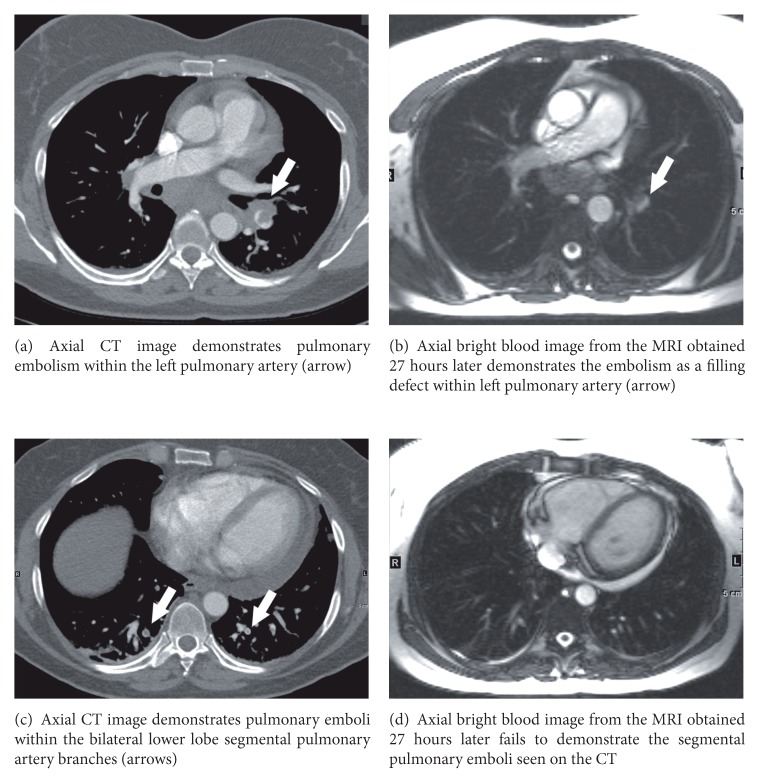
47-year-old female with left pulmonary artery and bilateral lower lobe segmental pulmonary artery emboli.

PE was identified within a total of 48 pulmonary arterial branches by CTPA (Table 1). Of these vessels, MRI detected emboli in 33, resulting in a per vessel sensitivity of 69%.  Figure 1 shows the typical appearance of pulmonary embolism on CT and MRI, while Figure 2 illustrates a patient with left pulmonary artery embolism detected by MRI, but bilateral lower lobe segmental branch emboli missed on MRI. Six patients had PE involving only lobar, segmental, or subsegmental pulmonary artery branches, while five patients had PE extending from the main, right, or left pulmonary artery. All eight pulmonary emboli located in the right or left pulmonary artery were detected on MRI. Of the 15 vessels containing PE that were not detected on MRI, 6 were within segmental branches, 7 were within subsegmental branches, and 2 were located in a subsegmental branch that was not included in the MRI field of view (Table 2). Of the 6 missed segmental pulmonary emboli, 2 could be identified retrospectively. Interobserver agreement was very good (*κ* = 0.93).

Of the 11 total patients, 9 had PE identified on MRI, for a per patient sensitivity of 82%. The two patients without identifiable PE by MRI had only segmental and sub-segmental emboli on CTPA. 

## 4. Discussion 

This study demonstrates the feasibility of diagnosing pulmonary embolism without the use of radiation or intravenous contrast media. Although our MRI protocol was not optimized for the detection of PE, it was still 69% sensitive on a per vessel and 82% sensitive on a per patient basis. 

Currently, CTPA is considered the gold standard for the diagnosis of PE [2, 3]. Unenhanced MRI has several potential advantages over CTPA. Perhaps most importantly, MRI does not require the use of ionizing radiation, while CTPA results in substantial patient radiation doses [5, 10–12]. This is of particular importance in young patients and patients who undergo repeated studies for the evaluation of possible PE. 

CTPA also requires the use of iodinated contrast media and, therefore, has the possibility of contrast reactions [4, 13–15] and exacerbation of renal function impairment. To date, the majority of studies evaluating the use of MRI in the diagnosis of PE have also used IV contrast [7], albeit gadolinium-based rather than iodinated contrast. Although the rate of serious acute adverse reactions to gadolinium-based contrast is lower than that to iodinated contrast [16–18], reactions do occur. Of even greater concern in patients with renal insufficiency is the association of gadolinium-based contrast agents with nephrogenic systemic fibrosis [19, 20]. The unenhanced MRI protocol for the detection of PE used in this study avoids the potential for any of these complications. 

Previous studies examining the use of MRI in the diagnosis of PE have evaluated noncontrast MRI only as part of a larger MRI protocol including contrast-enhanced MRI [20]. The largest such previous study demonstrated a per patient and per vessel sensitivity of 89% and 81%, respectively, for the detection of pulmonary embolism by noncontrast MRI, using CTPA as a reference standard. The sensitivities in our study are slightly lower, likely due to differences in our MRI protocol. A recent study evaluating several different MRI sequences in the detection of known pulmonary emboli demonstrated 67% sensitivity on a per vessel basis using a noncontrast sequence, results which are similar to our study [21]. 

An additional potential diagnostic tool in the evaluation of patients with suspected pulmonary embolism is single photon emission computed tomography ventilation/perfusion lung scan (V/Q SPECT). Although this modality avoids the use of iodinated or gadolinium-based contrast agents, it does still subject the patient to ionizing radiation. One recent study examining the utility of V/Q SPECT found that the prevalence of pulmonary embolism was 88% in patients with a high-probability V/Q SPECT exam [22]. This study did not use CTPA as the reference standard, however, making direct comparison without results difficult.

This study has several limitations. As mentioned above, the MRI protocol was not specifically designed for the evaluation of the pulmonary vasculature. An improved MRI protocol, using multiplanar imaging, decreased slice thickness and separation, increased field-of-view, and increased in-plane resolution would likely improve the sensitivity for detection of MRI. The study is also limited by the small number of patients. 

This study was also limited by the timing of the MRI. As MRI was not performed immediately following CTPA, it is possible that by the time of the MRI the emboli seen on the CTPA had been lysed, particularly given that our patients were treated with anticoagulation between the CTPA and the MRI.

An additional potential limitation of MRI for the evaluation of PE is the substantially longer time required for image acquisition. Although in this study all patients were able to complete the MRI, it did take significantly longer than the CTPA (55.6 minutes for MRI versus 2.7 minutes for CTPA, *P* < 0.001). Given this time difference, it is possible that some acutely ill patients may not be able to tolerate the MRI.

There is an inherent bias in our study design, in that all patients in the study we known to have PE on CTPA. Therefore when reviewing the MRIs the readers knew that PE should be present, this may have biased their interpretations of the exam. 

We conclude that noncontrast MRI offers a means of diagnosing PE without intravenous contrast material or radiation, although further study is necessary before it can be considered a routine tool in the evaluation of the patient with suspected PE. It is our hope that with the increased availability of MRI in the emergency setting and growing concern over radiation exposure, this technique will be used in patients with contraindications to ionizing radiation or intravenous contrast. 

## Figures and Tables

**Table 1:** Sensitivity, specificity, positive and negative predictive values for detection of pulmonary emboli by pulmonary artery distribution.

	Right				Left			
	Right pulmonary artery	Upper lobe	Middle lobe	Lower lobe	Left pulmonary artery	Upper lobe	Lingula	Lower lobe
Sensitivity	100 (4/4)	29 (2/7)	67 (4/6)	100 (8/8)	100 (4/4)	33 (2/6)	50 (2/4)	78 (7/9)
Specificity	100 (7/7)	100 (4/4)	100 (5/5)	100 (3/3)	100 (7/7)	100 (5/5)	100 (7/7)	100 (2/2)
Positive predictive value	100 (4/4)	100 (2/2)	100 (4/4)	100 (8/8)	100 (4/4)	100 (2/2)	100 (2/2)	100 (7/7)
Negative predictive value	100 (7/7)	44 (4/9)	71 (5/7)	100 (3/3)	100 (7/7)	56 (5/9)	78 (7/9)	50 (2/4)

**Table 2:** Distribution of pulmonary emboli not identified on MRI.

	Right upper	Right middle	Right lower	Left upper	Lingula	Left lower	Total
Lobar	0	0	0	0	0	0	0
Segmental	2	0	0	2	1	1	6
Subsegmental	2	2	0	1	1	1	7
Not covered	1	0	0	1	0	0	2
